# Green Synthesis of Zinc Oxide Nanoparticles Using Pomegranate Fruit Peel and Solid Coffee Grounds vs. Chemical Method of Synthesis, with Their Biocompatibility and Antibacterial Properties Investigation

**DOI:** 10.3390/molecules27041236

**Published:** 2022-02-12

**Authors:** Hala M. Abdelmigid, Nahed Ahmed Hussien, Amal Ahmed Alyamani, Maissa M. Morsi, Noha Moslah AlSufyani, Hanan Abdulaziz kadi

**Affiliations:** 1 Department of Biotechnology, College of Science, Taif University, P.O. Box 11099, Taif 21944, Saudi Arabia; h.majed@tu.edu.sa (H.M.A.); a.yamani@tu.edu.sa (A.A.A.); h.gadi@tu.edu.sa (H.A.k.); 2 Department of Biology, College of Science, Taif University, P.O. Box 11099, Taif 21944, Saudi Arabia; m.moasa@tu.edu.sa (M.M.M.); nohaalsufyani@hotmail.com (N.M.A.)

**Keywords:** ZnO nanoparticles, green, chemical, Vero E6, MTT, antibacterial activity

## Abstract

This research aims to investigate the synthesis, characterization, and evaluation of the biocompatibility and antibacterial activity of novel zinc oxide (ZnO) nanoparticles (NPs) prepared by *Punica granatum* peel and *coffee* ground extracts as the reducing and capping agents. Chemically synthesized ZnONPs were prepared using zinc acetate dihydrate and sodium hydroxide as reducing precursors. ZnONPs were characterized using an ultraviolet-visible spectrophotometer (UV-VIS), X-ray diffraction (XRD), scanning electron microscope (SEM), transmission electron microscope (TEM), and Fourier transform infrared (FTIR) spectroscopy. Peaks of UV spectra were 300 nm for ZnONPs_PPE, 320 nm (ZnONPs_CE), 290 nm, and 440 nm (ZnONP_Chem), thereby confirming ZnONPs formation. The X-ray diffractograms revealed their hexagonal structure. TEM micrographs of the biosynthesized ZnONPs revealed their hexagonal pattern and nanorod shape for ZnONPs_Chem with particle sizes of 118.6 nm, 115.7 nm, and 111.2 nm, respectively. The FTIR analysis demonstrated the presence of proteins, carboxyl, and hydroxyl groups on ZnONPs surfaces that act as reducing and stabilizing agents. ZnONP_Chem shows the antibacterial effect on *Staphylococcus aureus*, *Enterobacter aerogenes*, *Pseudomonas aeruginosa*, and *Klebsiella pneumoniae*. *Punica* peel and coffee ground extracts are effective reducing agents for green ZnONPs synthesis with a lower cytotoxic effect on Vero cells than ZnONPs_Chem with IC50 = 111, 103, and 93 μg/mL, respectively.

## 1. Introduction

Nanotechnology provides considerable significance in various fields due to the distinctive features of nanoparticles (NPs). One of the imperative applications of nanotechnology is in the field of plant science that affords beneficial effects to plants and soil by functioning as fertilizer to improve plant growth and productivity and as biosensors to monitor soil quality and plant health. Engineered nanomaterials could potentially interact with biomolecules and intracellular processes as many biological activities take place at the nanoscale level [1]. From this point of view, ZnONPs are of particular concern since they are believed to be non-toxic, safe, and biocompatible [2]. Additionally, ZnONPs have optical and electrical capabilities and catalytic and antimicrobial activities [3]. Lately, the use of ZnONPs in agriculture is arising as a prospective tool to plant science, providing promising aspects for higher quality plant growth and yield to help minimize reliance on chemical fertilizers for sustainable agricultural development and food security and fulfill the nutritional needs of the world’s fast-growing population [4,5,6]. In innovative agriculture, ZnONPs are investigated to achieve their sustainable development and assess their capability for promoting growth by examining them as nano-fertilizers in crops such as corn, onion, tomato, pepper, and wheat [7].

As the biological activities of ZnO nanoparticles are size and morphology dependent, the synthesis of ZnO nanomaterials of the desired size and shape is the subject of investigation by many researchers [8,9,10], and diverse types of ZnONPs shapes are recorded based on the process of synthesis. Approaches of NPs synthesis are divided into three categories: physical, chemical, and biogenic. Chemical synthesis is one of the most imperative techniques which can be accomplished by using a range of precursors and different conditions such as the concentration of reactants, time, temperature, and so forth. Fluctuation of these parameters leads to morphological variations in size and geometries of resulting nanoparticles. However, the chemical processes are associated with environmental pollution, high temperatures, high pressures, and expensive equipment [11]. Hence, there is a rising need for a simple, fast, and eco-friendly method to synthesize ZnONPs. Alternatively, biological approaches are increasingly used for nanoparticle fabrication [8,9,10]. They afford numerous advantages over the other techniques, including being clean, cost-effective, and having single-step protocols [8]. Furthermore, green fabricated NPs reveal distinguishing properties such as their optical, photo-electrical, and chemical characteristics, which give them the competence to be used for a wide range of applications, including agricultural purposes. In this context, the biological system will fulfill the function of biological laboratories for synthesizing metal oxide nanoparticles via a biomimetic approach.

Several plant extracts are being used in the green synthesis of nanoparticles because they contain several important metabolites and biomolecules that function as reducing, stabilizing, and capping agents for synthesizing NPs, including ZnONPs, which have improved the field of nanoscience [12,13,14]. Pomegranate (*Punica granatum* L.) is one of the most significant crops cultivated in the Taif region in Saudi Arabia. The fruit of this crop is rich in polyphenolic phytochemicals [15] and therefore is widely used in the food industry and in traditional medicine to treat various diseases [16]. Pomegranate peel is one of the major agro-wastes, which constitutes approximately 60% of the weight of pomegranate fruit [17,18]. Coffee (*Coffea arabica*) is one of the world’s most popular beverages that is commercially cultivated [19]. It is regarded as a rich source of biologically active compounds, particularly polyphenols [20]. The coffee industry is responsible for the production of vast amounts of waste. Solid coffee ground (SCG) constitutes (hemi) cellulose, proteins, melanoidins, and polyphenols (tannins, lignins, and chlorogenic acids) [21]. Polyphenol-rich aqueous extracts of arabica coffee beans such as chlorogenic acids and melanoidins may generate nanostructures through oxidative coupling.

Antimicrobial activities of metal oxide (ZnONPs) against pathogenic microorganisms that can cause diseases in plants and animals, including Gram-positive and Gram-negative bacteria (e.g., *Klebsiella pneumoniae, Staphylococcus aureus*, *Escherichia coli* and *Pseudomonas aeruginosa*) were quantitatively assessed in the culture media [22,23]. The detected reactive oxygen species (ROS) generated by these metal oxide particles could be the major mechanism of their antibacterial activity [24,25]. The antibacterial mechanism of ZnONPs encompasses the direct interaction between ZnO nanoparticles and cell surfaces affecting cell membrane permeability; then NPs enter and induce oxidative stress in bacterial cells, which cause the inhibition of cell growth and eventually cell death [26,27]. Verification of antibacterial activity of ZnONP is promoting its application in the seed preservation of crops during the storage period before cultivation.

Ethical and safety issues that surround the use of ZnONPs for agronomic applications should be considered and thoroughly evaluated. Considering possible nanoproducts in the food and agribusiness domains, scientific efforts to address risks are urgently needed for comprehensive evaluations of the environmental impact of nano fertilizers. Toxicity studies identify the risks associated with the bioaccumulation of NPs in the food chain and make it possible to design suitable fertilizer products with appropriate dosages for their effective use. Several studies suggested that ZnONPs toxicity mechanism encompasses the production of ROS [28,29,30] in biological systems in response to NPs diffusion, which may interfere with typical biophysicochemical and abiotic stress-related capabilities [31]. ZnONPs were investigated for toxicity on mammalian cells, and an increase in oxidative stress, cell membrane damage, and cytotoxicity was reported in various mammalian cell lines as the most frequent toxic effect of zinc-based nanomaterials [32]. Accordingly, the risk assessment of ZnONPs was determined by dose, exposure time, and cell type, regardless of the synthesis method [33,34]. However, many reports are concerned about the cytotoxic effects of ZnONPs on different cell lines. MTT assays were used for measuring cell viability. Using Vero E6 cells as a model and MTT as an assay technique, previous reports clearly suggested that the cytotoxicity of ZnONPs was dependent on concentration, exposure time, cell type, and proliferation, irrespective of the synthesis approach [35,36].

To our knowledge, the green fabrication of ZnONPs using agro-industrial wastes and their antimicrobial and cytotoxicity properties are not adequately documented. In this perspective, the main objective of this study was to investigate the use of pomegranate peel (PP) and solid coffee ground (SCG) extracts as capping agents for the in vitro synthesis of ZnONPs and evaluate their cytotoxicity on normal Vero E6 cell lines using an (MTT) assay. The antibacterial activity against Gram-positive (methicillin-resistant *Staphylococcus aureus*) and Gram-negative bacteria (*Escherichia coli; Klebsiella pneumoniae, Pseudomonas aeruginosa*) was performed using the agar disc diffusion method. Overall, this study might be considered an initial prerequisite step to agri-nanotechnology realistic research in the Taif area.

## 2. Materials and Methods

### 2.1. Cells, Bacterial Strains, and Chemicals

The metal oxide precursor, zinc acetate dihydrate (Zn(C_2_H_3_O_2_)_2_.2H_2_O), was purchased from Sigma-Aldrich, Saint-Louis, MO, USA. The bacterial strains used in this study were: Methicillin-resistant *Staphylococcus aureus* (ATCC 29213), *Enterobacter aerogenes* (ATCC™ 35029™), *Pseudomonas aeruginosa* (ATCC 27853), and *Klebsiella pneumoniae* (BAA-2473™). Vero E6 (ATCC^®^ CRL-1586™) was obtained from the American Type Culture Collection (ATCC).

### 2.2. Biosynthesis of ZnO Nanoparticles

**Figure 1:**
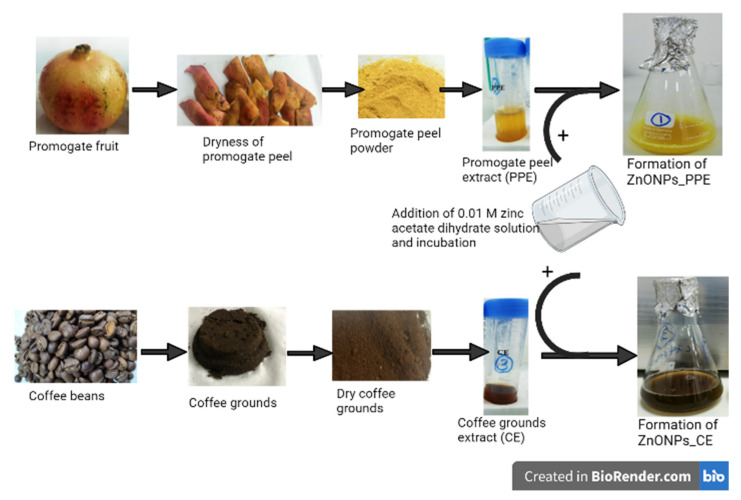
Schematic illustration of ZnONPs preparation using pomegranate peel extract (PPE) and coffee ground extract (CE). Created in BioRender.com.

For green fabrication of ZnONPs, extract preparation of *Punica granatum* L. peel (PPE) and coffee grounds (CE) was carried out in accordance with a previously described study by Abdelmigid et al. [37]. Briefly, 95 mL of 0.01 M zinc acetate dihydrate solution (Zn(C_2_H_3_O_2_)_2_.2H_2_O) was mixed with 5 mL of each extract. Mixtures were incubated for 1 h (hour) at 70 °C with continuous stirring. pH was adjusted to alkaline (pH = 10) by NaOH addition. After the incubation period, the powdered precipitates were formed in the mixtures (Figure 1), then centrifuged for 30 min. at 3000 rpm. The supernatant was decanted, and the precipitate was thoroughly washed with distilled water. Finally, pellets were transferred to Petri dishes for complete dryness at 60 °C overnight [38].

### 2.3. Chemical Synthesis of Zinc Oxide Nanoparticles

Zinc acetate dihydrate (2 g) was dissolved in distilled water (15 mL), while sodium hydroxide (NaOH, 8 g) was dissolved in 10 mL of distilled water. Both solutions were stirred separately for 5 minutes at room temperature. After mixing, a solution of NaOH was added to zinc acetate dihydrate solution with a continuous stirring for 5 min. Then, 100 mL of ethyl alcohol was added to the previous mixture drop by drop. Finally, a white precipitate of ZnO nanopowder was formed [39].

### 2.4. Characterization of Zinc Oxide Nanoparticles

The UV-VIS spectroscopy analysis was conducted using a UV–VIS-NIR spectrophotometer (UV-1601, Shimadzu, Kyoto, Japan) at a wavelength ranging from 200–800 nm to confirm the manufacture of ZnONPs, and deionized water was utilized as a blank. Absorption spectra of the biologically synthesized zinc oxide nanoparticles (ZnONPs_PPE, and ZnONPs_CE) and the chemically synthesized ZnO nanoparticles (ZnONPs_Chem) were measured in their aqueous form before dryness. The morphology, size, and shape of different ZnO nanoparticles (ZnONPs_PPE, ZnONPs_CE, and ZnONPs_Chem) were determined by scanning electron microscope (SEM) (JSM-639OLA, JEOL, Tokyo, Japan), transmission electron microscope (TEM) (JSM-1400 PLUS, JEOL, Tokyo, Japan), and X-ray diffractometer (XRD) (Pan Analytical, X-pert pro, Almelo, Netherland). The surface morphology of the samples was studied by SEM performed on (JSM-639OLA, JEOL, Tokyo, Japan) at 20 KV with various magnifications ×500 (scale bars = 50 μm), ×2000 (scale bars = 10 μm), and ×3000 (scale bars = 5 μm). Dry NPs powder were coated with gold for 10 min using Cressington Sputter Coater (108auto, thickness controller MTM-10, Essex, UK). The particle shape and size were determined by Transmission Electron Microscopy (TEM), carried out on JEOL– JSM-1400 PLUS (JEOL, Tokyo, Japan) at 100 kV. The phase of synthesized ZnO nanopowders was characterized using XRD at 30 kV and 100 mA, and the spectra were recorded with CuKα radiation (λ = 1.5406 Å) in the 2θ (20°–80°). Patterns of XRD were plotted by OriginLab software^®^ (2018) and compared with JCPDS Card No. 36-1451.

Dynamic light scattering (DLS and Zeta analyses of ZnO NPs were performed using a particle size analyzer (Zetasizer Nano ZS, Malvern Instruments Ltd., Malvern, UK) to investigate the particle size and surface charge of the prepared ZnO nanoparticles (detection angle = 90°). The electrostatic potential of the particles was determined using an ultrasonic dispersion of freshly resuspended ZnONPs powder in 0.9% saline solution at high speed (30 min at 25 °C) to prevent their aggregations before evaluation. To determine the functional groups that are responsible for ZnONPs formation and stabilization, Fourier Transforms Infrared Spectroscopy (FTIR, Agilent technologies, Santa Clara, CA, USA) was used over a wavelength range of 450 to 4000 cm^−1^.

### 2.5. Biocompatibility Assay

#### 2.5.1. Cell Lines and Culture Conditions

Normal monkey kidney cell line, Vero E6, was cultured into 75 cm^2^ flasks in DMEM-high glucose media (Sigma-Aldrich, Taufkirchen, Germany) supplemented with 4500 mg/L glucose, 2 mM L-glutamine, 1 mM sodium pyruvate, 10% fetal bovine serum (FBS), and antibiotics (penicillin 100 IU/mL, streptomycin 100 µg/mL). The cell culture was kept under standard culture conditions (37 °C, 95% humidified air, and 5% CO_2_).

#### 2.5.2. MTT Assay

Further, Vero E6 cells were cultured in 96-well culture plates in DMEM-high glucose (Sigma) supplied with 10% FBS at a concentration of 1 × 10^5^ cells/mL. After 24 h, the culture medium was discarded and then the cells were treated with ZnONPs_PPE, ZnONPs_CE, and ZnONPs_Chem (serial concentrations 40, 60, 80, 100, 120, 140, 160, 180, 200 μg/mL) in serum-free media, separately. After 24, 48, and 72 h of incubation, the culture media were discarded, and then 50 µL of 3-(4,5-Dimethylthiazol-2-yl)-2,5-diphenyltetrazolium bromide (MTT, 0.5 mg/mL PBS) was added per each well. The cells were incubated for 4 h (37 °C, 95% humidified air, and 5% CO_2_) then 50 µL of DMSO was added per each well. Afterwards, the plates were shaken for 10 min, and absorption was measured using an ELISA microplate reader at wavelength 570 nm [40]. The media without any NPs was used as a negative control, and each concentration was assayed in triplicate. The effect of ZnONPs was quantified as the percentage of control absorbance of reduced dye at 570 nm. The following equation was utilized to calculate cell viability (percentage):

Cell viability (%) = Mean OD of treated cells/Mean OD of control (untreated cells) × 100

Data were plotted by using the Microsoft Excel program. The IC_50_ values (50% inhibition concentration) for different prepared ZnONPs at different sampling times were determined using the IC50 Calculator of AAT Bioquest ^®^ 2019.

### 2.6. Antibacterial Activity

Disk diffusion method was undertaken to evaluate in vitro antibacterial activity of the synthesized green and chemical ZnO nanoparticles against four different pathogenic strains: They include Gram-positive (methicillin-resistant *Staphylococcus aureus* (MRSA)) and Gram-negative (*Enterobacter aerogenes*, *Pseudomonas aeruginosa*, and *Klebsiella pneumoniae*) bacteria using the disk diffusion method. Briefly, Mueller–Hinton agar plates were inoculated with each pathogenic bacterial strain, separately. Wells with a diameter of 7 mm were cut using sterile blue tips. For each plate, four wells were created, one for plant extract (100 μL of PPE or CE), and three different concentrations of each ZnO nanoparticle (100 μL of 2 mg/mL, 4 mg/mL, and 8 mg/mL), respectively. Ciprofloxacin (Accord, UK) (3 mg/mL, 100 μL/well) was used as a positive control. Plates were incubated overnight at 37 °C. After incubation, the inhibition zone diameter was measured in mm using a standard metric ruler.

### 2.7. Statistical Analysis

Data were expressed as the mean ± standard error (M ± SE). Statistical analysis was conducted using a one-way ANOVA to differentiate between the inhibition zone diameter of different bacterial strains groups within the same ZnO treatment using GraphPad software (GraphPad, 2017)^®^. In which, *** indicates *p* ≤ 0.001, ** indicates *p* ≤ 0.01, * indicates *p* ≤ 0.05 and ns (non-significant) means *p* > 0.05.

## 3. Results

### 3.1. Characterization of Zinc Oxide Nanoparticles

#### 3.1.1. Synthesis of ZnONPs and UV–Vis Spectroscopy Profile of ZnONPs

**Figure 2:**
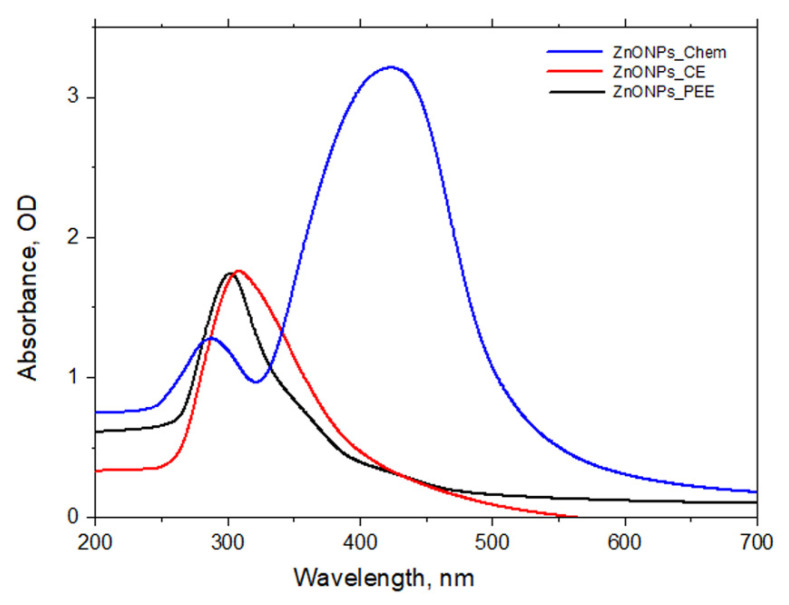
UV-Visible spectral analysis of green synthesized ZnONPs_PPE, ZnONPs_CE, and chemically synthesized ZnONPs_Chem.

Initial confirmation of ZnONPs formation was realized by visual observation of the reaction mixture. This is indicated by the appearance of white precipitates of ZnONPs_Chem deposited at the bottom of flasks. Meanwhile, the green-mediated synthesis was also checked by observing color changes in the reaction suspension. Prior to incubation, the color of plant extracts (PPE and CE) in distilled water was clear, and after the addition of Zn^2+^, the color of the reaction mixture changed to dark yellow and dark brown, respectively, implying the reduction of Zn^2+^ to ZnONPs (Figure 1). After the recovery of ZnONPs from the chemical and biological path, the obtained white powder was used for further analysis. The reduction of Zn^2+^ to ZnONPs was assessed by UV–Vis spectroscopy of the colloidal solution in the range of 300–700 nm. The UV–Vis spectrum of ZnONPs_PPE and ZnONPs_CE samples showed a profound peak at 300 and 320 nm, respectively, which is the typical characteristic of ZnONPs and confirmed the formation of ZnONPs. Meanwhile, ZnONPs_Chem showed two peaks, one broad at 290 nm and one sharp at 440 nm (Figure 2).

#### 3.1.2. Morphology, Size, and Shape of ZnONPs

**Figure 3:**
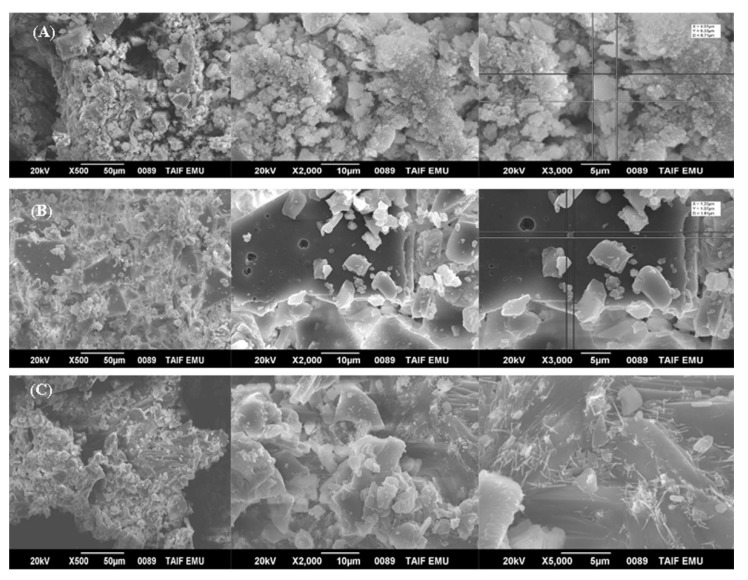
Scanning electron microscope photos showing the crystalline shape of ZnONPs_PPE (**A**), ZnONPs_CE (**B**), and ZnONPs_Chem (**C**) with different magnifications. In addition, at higher magnification, ZnONPs_Chem preparation has nanorods.

Surface morphology of the biosynthesized ZnONPs_PPE and ZnONPs_CE and chemical mediated ZnONPs_Chem were studied using the SEM. The SEM micrographs are shown in Figure 3A–C), respectively. The SEM analyses revealed the presence of agglomerates of the nanoparticles. The micrographs showed that the nanocrystals of uncalcined ZnONPs_PPE and ZnONPs_CE are interlinked to one another to make large network systems with irregular pore sizes and shapes. SEM images of ZnONPs_Chem showed the formation of chunky particles of non-homogenized nanoparticles, while nanorods significantly appeared at high magnification (×5000).

**Figure 4:**
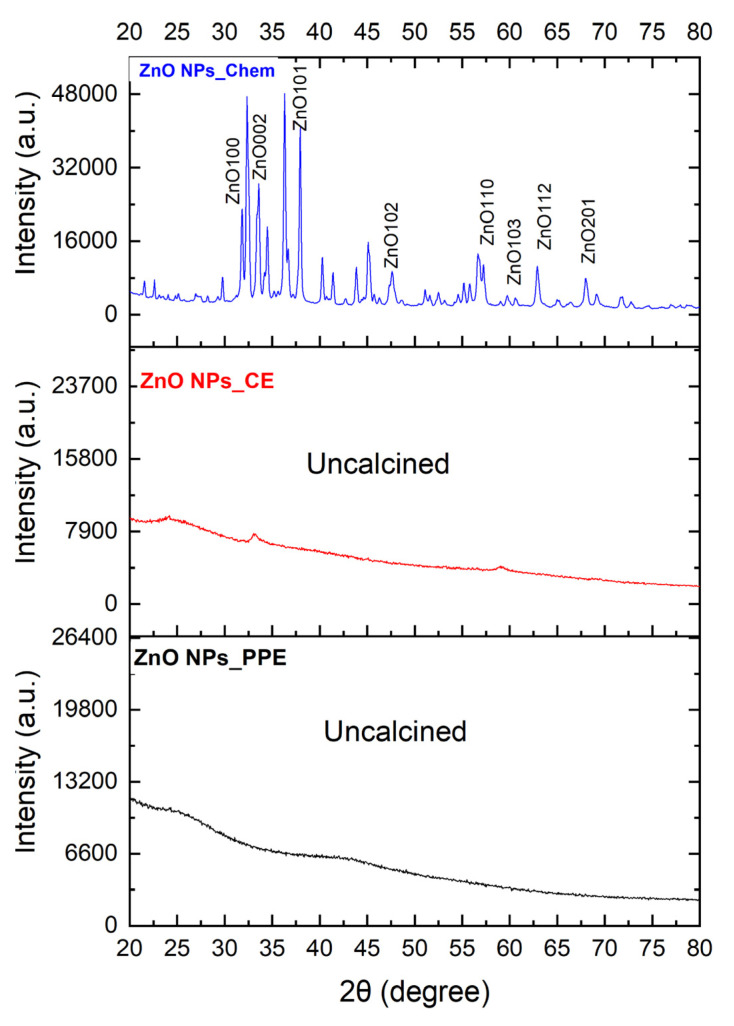
XRD patterns of ZnONPs_PPE, ZnONPs_CE, and ZnONPs_Chem. ZnONPs_Chem shows sharp peaks than the biologically synthesized uncalcined zinc nanoparticles.

The XRD profile of ZnONPs_Chem showed sharp and distinct peaks of (2θ) 31°, 35°, 37°, 48°, 56°, 62°, 68°, and 69°, which were indexed as the planes 100, 002, 101,102, 110, 103, 112, and 201 (Figure 4). These peaks are well coordinated with wurtzite ZnO from the Joint Committee on Powder Diffraction (JCPD) standards, card number (36–1451). Thus, the XRD pattern revealed that ZnONPs_Chem with a fine hexagonal crystalline structure developed in close accordance with this reference model. Other diffraction peaks could be due to impurities of unreacted zinc. Conversely, the uncalcined ZnONPs_PPE and ZnONPs_CE show unsharp low peaks (Figure 4).

**Figure 5:**
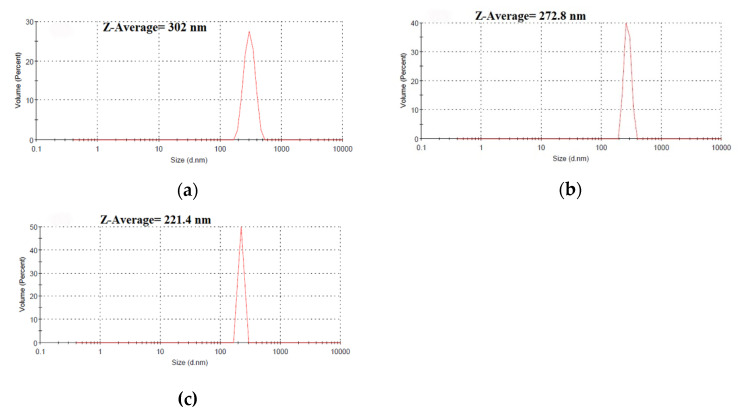
Average size of green synthesized (**a**) ZnONPs_PPE (302 nm), and (**b**) ZnONPs_CE (272.8 nm), and chemically synthesized (**c**) ZnONPs_Chem (221.4 nm).

**Figure 6:**
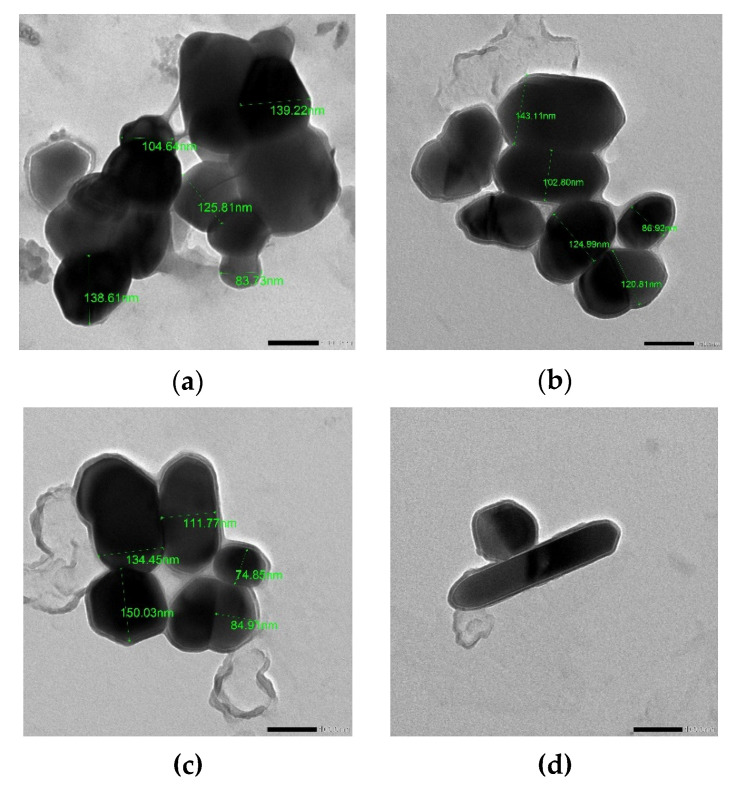
TEM images of ZnONPs_PPE (**a**), ZnONPs_CE (**b**), and ZnONPs_Chem (**c**) showing their hexagonal crystalline shape, in addition to the nanorod shape of ZnONPs_Chem (**d**).

The hydrodynamic size of synthesized ZnONPs was characterized using the DLS technique. The Z-average size of ZnONPs_PPE = 302.0 nm, ZnONPs_CE = 272.8 nm, and ZnONPs_Chem = 221.4 nm with a zeta potential −11.7 mV, −13.0 mV, and −17.9 mV, respectively (Figure 5). The TEM images of ZnONPs are shown in Figure 6a–d. The TEM study was conducted to understand the crystalline characteristics and size of the nanoparticles. The images of all ZnONPs confirm the hexagonal crystalline shape in accordance with XRD data. Moreover, the nanorod shape of ZnONPs_Chem (Figure 6d) was also confirmed as revealed by SEM micrograph (x = 5000). The average particle size was found to be 118.6 nm, 115.7 nm, and 111.2 nm for ZnONPs_PPE, ZnONPs_CE, and ZnONPs_Chem, respectively. Overall, it is observed that the TEM images revealed a smaller particle size compared to that obtained from the DLS analysis.

#### 3.1.3. FTIR analysis

**Figure 7:**
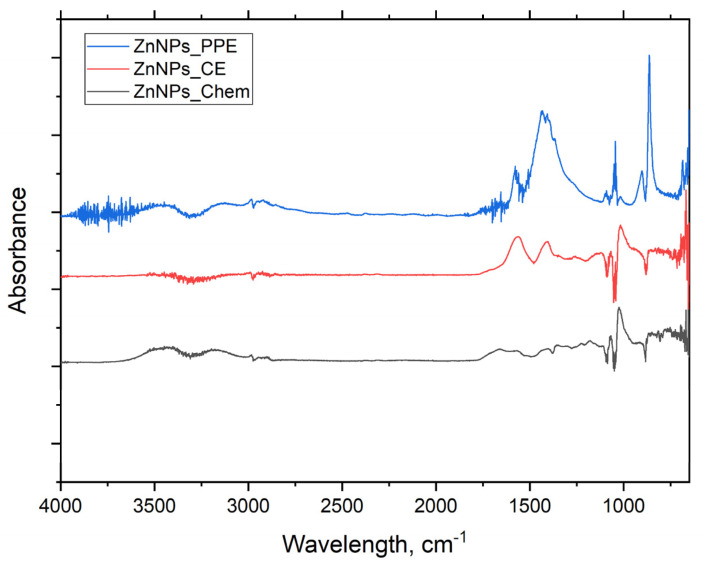
FTIR spectra of green ZnONPs_PPE, ZnONPs_CE, and chemically synthesized ZnONPs_Chem.

FTIR spectroscopy helps to identify the functional groups that participated in the nanoparticle’s synthesis and their distribution on the resulting ZnONPs. Figure 7 illustrate the FTIR absorption spectra within the range of 4000 to 450 cm^−1^. Broad absorption peaks are found at 3350, 1570, 1425, 1350, 1233, 1027, 675, 648, 613, and 581 cm^−1^ for ZnONPs_PPE. The FTIR spectra of ZnONPs_CE powder displayed numerous absorption peaks at 3400, 1600, 1400, 1000, 480–450 cm^−1^. On the other hand, ZnONPs_Chem showed fewer broad absorption peaks at 3500 and 1700–1500 cm^−1^, but weak peaks were also noticed at 500 cm^−1^.

### 3.2. Biocompatibility of Synthesized ZnONPs

**Figure 8:**
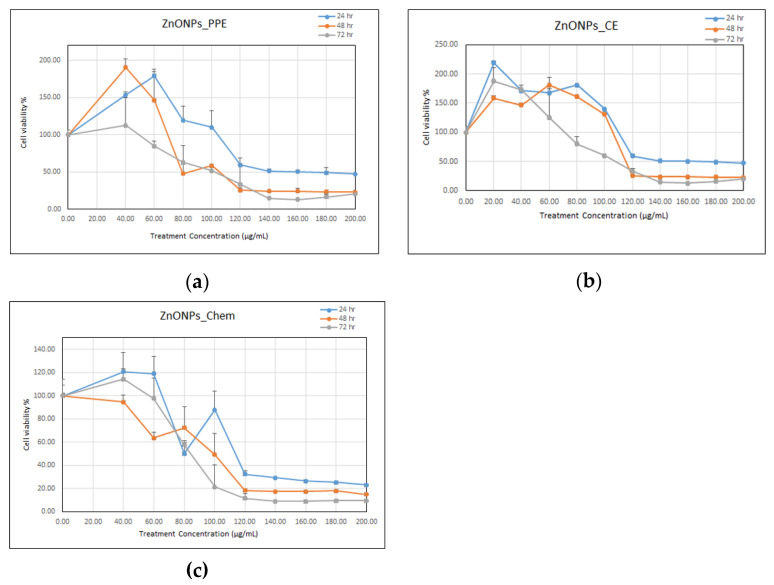
Percentage of cell viability of Vero E6 cells according to serial dilutions treatment of ZnONPs_PPE (**a**), ZnONPs_CE (**b**), and ZnONPs_Chem (**c**) at different sampling times 24 h, 48 h, and 72 h.

MTT assay was utilized to investigate the cytotoxic effects of the biosynthesized ZnONPs_PPE and ZnONPs_CE as well as the chemical-mediated ZnO nanoparticles (ZnONPs_Chem) on the Vero E6 cell line. Based on the results in Figure 8a–c, ZnONPs exhibited cytotoxic effects against Vero cells in a time and dose-dependent manner. After 72 h, cell viability decreased significantly at the concentrations of 100, 120, 140, 160, 180, 200 μg mL^−1^ when treated with ZnONPs_PPE. Another pattern was observed for ZnONPs_CE, whereby cell viability decreased significantly at concentrations 60, 100, 120, 140, 160, 180, 200 μg/mL. ZnONPs_Chem had a more distinctive cytotoxic effect as cell viability decreased significantly in a steady manner at concentrations 40, 60, 80, 100, 120, 140, 160, 180, 200 μg/mL, after 72 h exposure.

The cytotoxicity was verified as the NPs concentration that causes 50% growth inhibition (IC50) of the cell line. After 24, 48, and 72 h exposure, the IC50 value of ZnONPs_PPE on Vero cells occurs at 133, 107, and 93 μg/mL, respectively. A similar pattern was exhibited by the ZnONPs_CE, which revealed IC50 values of 120, 100, and 90 μg/mL after 24, 48, and 72 h, respectively. The highest cytotoxicity levels on Vero cells were exhibited by ZnONPs_Chem as they recorded IC50 values at 107, 91, and 82 μg/mL after 24, 48, and 72 h, respectively. Overall, the average IC50 for ZnONPs_PPE, ZnONPs_CE, and ZnONPs_Chem were 111, 103, and 93 μg/mL, respectively.

### 3.3. Antibacterial Activity of ZnO Nanoparticles

**Figure 9:**
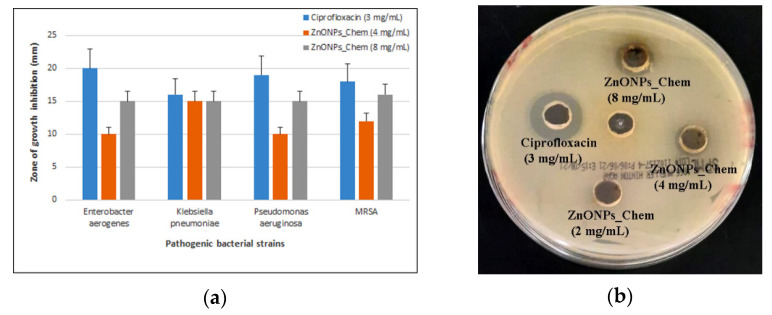

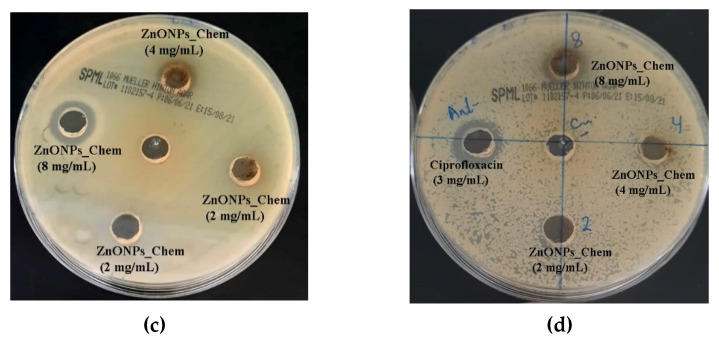
Antibacterial effect of chemically synthesized ZnONPs_Chem (4 mg/mL and 8 mg/mL) in comparison to Ciprofloxacin (3 mg/mL) drug on different pathogenic bacterial strains *Enterobacter aerogenes, Klebsiella pneumoniae, Pseudomonas aeruginosa*, and MRSA (**a**). Representative photographs of ZnONPs_Chem (2, 4, and 8 mg/mL) potential on different bacterial cells that show inhibition zones; *Enterobacter aerogenes* (**b**), *Pseudomonas aeruginosa* (**c**), and MRSA (**d**).

The in vitro antibacterial efficacy of ZnONPs was evaluated in a broad spectrum of pathogenic bacteria, including *Pseudomonas aeruginosa* and *Klebsiella pneumoniae*. In this study, the antibacterial activity of synthesized ZnONPs against Gram-positive (MRSA) and Gram-negative (*Enterobacter aerogenes*, *Pseudomonas aeruginosa*, and *Klebsiella pneumoniae*) bacteria were conducted using the agar well diffusion method. After incubation at 37 °C for 24 h, 100 μL of PPE and CE extracts did not show any inhibition on different bacterial strains, whereas ZnO nanoparticles demonstrated antibacterial efficacy with variable levels of activity. The biogenic ZnONPs (PPE and CE) did not show any antibacterial activity at the applied concentrations (2 mg/mL, 4 mg/mL, and 8 mg/mL). Conversely, ZnONPs_Chem exhibited a notable increase of inhibitory effect at higher concentrations (4 mg/mL and 8 mg/mL). A non-significant increase of the inhibitory zone was found at 8 mg/mL concentration in comparison to 4 mg/mL for all treated pathogenic bacteria. However, Ciprofloxacin (3 mg/mL) has a non-significant higher antibacterial effect than chemically synthesized zinc oxide nanoparticles. The average value of the inhibitory zone at different concentrations of ZnONPs against the tested pathogens is presented in Figure 9.

## 4. Discussion

Recently, the exploitation of plant extracts in the synthesis of NPs has gained considerable attention as an alternative to chemical and physical methods. The utilization of bioactive substances from plants leads to the elimination of expensive and harsh chemicals. The synthesis process of NPs can be achieved using extracellular or intracellular biological compounds from plants. In this study, the aqueous Zn^2+^ was reduced to ZnONPs when added to plant extracts derived from pomegranate peel extract (PPE) and coffee ground extract (CE). The reduction of Zn^2+^ to ZnONPs was proposed to occur by the action of biological compounds secreted into the reaction mixture by plant extracts and their functional group. Preliminary confirmation of ZnO nanoparticles formation is detected after adding plant extracts to zinc acetate dihydrate and incubating at 70 °C. Change in the color of the reaction mixture could be considered as an initial signature to ZnONPs biological formation. The phenolic compounds and flavonoids that are present in the selected plant extracts are thought to be responsible for ZnO reduction. ZnONPs_PPE and ZnONPs_CE synthesis was strongly proven by the UV-VIS spectroscopy at 300 nm and 320 nm, respectively. Likewise, prior studies of ZnONPs’ biosynthesis employing plant extracts (e.g., *Cassia fistula*, *Melia azadarach*, *Punica granatum*) reported the same range of absorption peaks [38,41]. On the other hand, the characteristic spectrum with two peaks in the absorption points at 290 nm and 440 nm for ZnONPs_Chem concur with Khoza et al. [42], who have successfully synthesized chemical-mediated ZnO nanoparticles utilizing zinc nitrate hexahydrate and sodium hydroxide as precursors, in the presence of ethanol.

As described by earlier studies, the spectrum of ZnO metal NPs shows the property of surface plasmon resonance which can shift in the wavelength due to the particle size or capping of phytochemicals from plant extract. Thus, the difference in absorbance of each prepared ZnONPs could be attributed to the difference in their size, as larger size NPs have lower absorbance, which in turn increases its Rayleigh scattering [43]. In this study, the chemical-mediated synthesis of ZnONPs could be attributed to the equilibrium between hydrolysis and condensation reactions [39], where zinc acetate was hydrolyzed to acetate and zinc ions during heating. Then, electrons of the abundant oxygen atoms promote bonding of the ethanol hydroxyl group with the zinc ions producing ZnO nanoparticles. Hasnidawani et al. [39] suggested the following formula to clarify the chemical synthesis of the ZnONPs.

(Zn (CH3COO)2.2H2O) + 2NaOH → ZnO (nanopowder) + 2NaCH3COO + H_2_O

The SEM, TEM, XRD, and DLS were undertaken to detect the structure, size, and surface potential of the produced ZnONPs. The current study’s results verified the formation of crystalline metal nanostructures and gave more insight into the hexagonal shape and size features of the metal nanoparticles [43]. TEM and SEM images showed that the produced biogenic ZnO-NPs (PPE and CE) were hexagonal nanocrystalline structures indicating that the used extracts of pomegranate fruit peel and the solid coffee ground had good capping and stability capabilities. The crystalline shape of the green synthesized ZnONPs was also reported in recent studies [38]. Moreover, ZnONPs_Chem showed distinct nanorod shaped crystals, which could be attributed to the elongation of the high energy 0001 plan of ZnO, which causes the conversion of spheres to nanorods during the chemical synthesis [44].

The XRD profile of the investigated ZnONPs_Chem showed significant intensity and small width for the diffraction peaks, indicating the good crystallinity of the final product. The hexagonal nanocrystalline formation with wurtzite structures of ZnO nanoparticles was in good accordance with the Joint Committee on Powder Diffraction (JCPD) standards, card number (36-1451), and prior studies. Conversely, the XRD spectrum of uncalcined biologically synthesized ZnONPs mediated by PPE and CE showed sharpless peaks compared to ZnONPs_Chem. This finding was previously reported by Bagabas et al. [45], who monitored low peak intensity in the XRD pattern of the uncalcined synthesis of ZnONPs with hexagonal nanocrystalline shapes.

In the same context, both DLS and TEM analyses revealed variation in size determination of ZnONPs depending on their preparation route and the used extract. The size of ZnONPs produced in this study could be ranked as follows: ZnONPs_PPE > ZnONPs_CE > ZnONPs_Chem. In a previous report [46], NPs size is anticipated as inversely correlates with surface negativity, where decreasing surface negativity leads to reduction of repulsion force and stability of nanoparticles, causing them to aggregate into large-sized particles. Looking at the surface negativity values in this research, it is clearly observed that the smaller sized ZnONPs_Chem revealed higher surface negativity compared to the biological synthesized ZnONPs (PPE and CE). Surprisingly, the size of the ZnONPs was greater in the DLS analysis compared to that detected by TEM. This might be attributed to the coating agent that capped and stabilized the surfaces of the NPs [46]. Furthermore, the non-homogeneous distribution of ZnO nanoparticles in the colloidal solution might explain the larger size in the DLS analysis. On the contrary, XRD analysis in prior studies recorded smaller sized ZnONPs synthesized from PPE (20–30 nm), CE (25.4–31.3 nm), and chemically mediated ZnO nanoparticles (25.25 nm) [47,48,49]. Different factors of the fabrication method such as pH, precursor concentration, temperature, and growth time have a significant impact on ZnO nanostructures [50] during ZnO nanoparticles synthesis which in turn precisely control their size, shape, surface architecture, and their properties in different practical fields. It is worth mentioning that, using the calcination approach for ZnONPs synthesis to reduce their final size [44], in this study, we employed a novel approach for synthesizing uncalcined ZnONPs.

Based on the results of the FTIR spectra of synthesized ZnONPs, the presence of hydroxyl functional, carbonyl, and ethylene groups may indicate the presence of carbohydrates and/or alcohols, ketone and/or quinones and alkene compounds. Here, the FTIR spectrum of ZnONP_PPE shows a band at 3350 cm^−1^, which is assigned to the O-H stretch of the polyphenol groups of plant extract [51]. Peaks at 1570 and 1425 cm^−1^ were attributed to the methylene vibration from protein and stretching of C-C in the aromatic groups, respectively [52]. The sharp peak at 1350 cm^−1^ indicates C-N stretch of the aromatic amines and the carboxylic acid, while the peak at 1027 cm^−1^ could be assigned to C-O stretching vibrations of alcohols, esters, ethers, C-N stretching of aliphatic amines, and carboxylic acids [53]. The lower band and peak at 891 and 690 cm^−1^ represent the C-H of aromatics, N-H of primary and secondary amines, and alkyl halides C-Br stretch, respectively [41]. The metal oxides also had lower absorption peaks which were caused by interatomic vibrations and represented a fingerprint of ZnO nanoparticles. The band absorbed at 613 and 580 cm^−1^ is characteristic of the Zn-O bond, which confirms the formation of zinc oxide [53,54]. The spectra observed between 500 and 900 cm^−1^ are related to metal–oxygen formation, whereas the ZnO stretching and vibration was attributed to peaks at 1634.00 and 620.93 cm^−1^ [47].

The FTIR spectra of uncalcined ZnONPs_CE displayed several absorption peaks that reflect the organic components present in CE. The broad band around 3400 cm^−1^ could be assigned to the O-H stretch of different carboxylic acids such as citric, chlorogenic, and caffeic acid, or the phenolic group of coffee ground [55]. The peak at 1600 cm^−1^ could be related to the asymmetric stretching vibration of -COOH or C=C ring stretching in polyphenols. On the other hand, the peak at 1400 cm^−1^ was attributed to the symmetric stretching vibration of the -COOH functional group or the (in-plane) bending vibrations of -OH in phenols. The peak around 1000 cm^−1^ represents the C-O stretching vibration [56,57,58]. Therefore, the presence of different functional groups of PPE and CE, such as alcohols, phenols, polyphenols, amines, carboxylic acids, ketones, and alkenes, may have contributed to Zn^2+^ biological reduction to ZnONPs_PPE and ZnONPs_CE, respectively. Moreover, carboxylic and phenolic acid groups are responsible for bio-capping, while free amino and carboxylic groups are responsible for synthesized zinc oxide nanoparticles stability during synthesis [41,59]. The FTIR spectra of the chemically synthesized ZnONPs_Chem show a broad band at 3500 assigned to the O-H stretch of water molecules present on the surface of nanoparticles. While the broadband at 1700–1500 cm^−1^ was assigned to the symmetric and antisymmetric C-O stretching vibration. Weak peaks around 500 cm^−1^ related to Zn-O bond secondary vibration, which indicates the well crystallization of the nanoproduct [49]

ZnO nanoparticles are multifunctional elements that are commonly used in many applications. However, its biocompatibility with biological organisms is a considerable concern. ZnONPs are reported to exert cytotoxic effects on various cell lines. Nevertheless, the cytotoxic effects are extremely reliant on many parameters, including the characteristics of NPs (size and shape), synthesis methods, as well as the type of cell line utilized. Consequently, most of the research has focused on reducing their toxic effects and improving their capabilities. In this study, the MTT assay was conducted to investigate the cytotoxic effects of the biosynthesized ZnONPs (PPE and CE) and chemical-mediated NPs (ZnONPs_chem) on the Vero E6 cell line. A distinct high cytotoxic effect was demonstrated by the ZnONPs_Chem, which might be due to the nanorod shape of the NPs created by this method. It was reported that the cytotoxicity of NPs is affected by their shape, size, and surface charge. The sharp nanostructures of the nanorod-shaped NPs exhibited a strong ability to damage the cell membrane, which subsequently causes cell death. A prior study reported that nanorod ZnO nanoparticles are more toxic than the other corresponding shapes with the same nanoparticle size and surface area [60]. Concerning risk assessment of ZnO nanoparticles based on MTT assay, it could be deduced that the produced biogenic ZnONPs in this study are safer for applications on biological systems. Less toxicity of ZnONPs_PPE and ZnONPs_CE could be assigned to their larger size compared to ZnONPs_Chem, and the presence of phytochemicals during the synthesis procedure on nanoparticles surfaces. These findings are consistent with an earlier study by Majeed et al. [61], who recorded less cytotoxic activity of biologically synthesized ZnONPs using leaves of *Artocarpus heterophyllus* on Vero cells. On the other hand, the nanorod shape of ZnONPs_Chem could easily enter the cell membrane and exert its toxic effect more than others [60].

The presence of the inhibition zone indicates the biocidal action of ZnO nanoparticles which involves disruption of the membrane due to the high rate of ROS generation and finally lead to the death of pathogens. Previous studies registered varying degrees of antibacterial potential according to the type of pathogens, synthesis method, and concentrations of ZnO nanoparticles [62]. As shown in this study, ZnONPs_chem demonstrated antibacterial efficacy on the tested strains while no sign of inhibition was detected by both green synthesized ZnONPs. As mentioned previously, in biocompatibility evaluation on Vero cells, the ZnONPs with a smaller size have a larger surface to volume ratio, and consequently, higher antibacterial efficacy due to their direct interaction with the bacterial cell membrane [62]. In addition, ZnONPs_Chem showed slightly higher antibacterial activity (a higher zone inhibition at 8 mg/mL) against the Gram-positive bacteria MRSA compared to the Gram-negative bacteria (*Klebsiella pneumoniae, Enterobacter aerogenes*, and *Pseudomonas aeruginosa*) in line with a previous study [63]. Sirelkhatim et al. [2] confirm that Gram-positive bacteria are more susceptible to ZnONPs inhibition than the Gram-negative bacteria that could be attributed to variations in their cell wall constitution, cell physiology, and metabolism [64,65]. ZnONPs_Chem efficacy against pathogenic bacterial strains could be attributed to oxidative stress caused by ROS generation, which leads to bacterial DNA damage [66]. Nevertheless, other researchers [63,67] verified the antibacterial potential of biogenic ZnONPs, utilizing higher concentrations of ZnONPs_PPE (up to 32 mg/mL) and ZnONPs_CE (300, 450, and 600 mg/mL) as opposed to lower concentrations applied in this study. Yet, initial insights into the antibacterial activity of our green ZnONPs product were established in this study. Therefore, we believe an antibacterial activity determination could be further established by applying higher concentrations.

## 5. Conclusions

In this study, biosynthesis of ZnONPs was successfully synthesized from extracts of agro-industrial wastes or residues such as pomegranate fruit peel (PPE) and solid coffee ground (CE) via a novel, simple, cost-effective, eco-friendly, and green approach and compared to the chemical-mediated synthesis method. Current results verified that PPE and CE could potentially be used as an effective reducing and capping agent for the biological synthesis of ZnONPs without having to use a calcination method. The synthesized ZnONPs were characterized using techniques such as UV-VIS XRD, SEM, TEM, DLS, and FTIR. The crystallinity of the biogenic ZnONPs was proved from the XRD analysis, and the analysis showed that all the diffraction peaks fit well with the hexagonal wurtzite structure. Furthermore, SEM and TEM analysis showed that the morphology of the biosynthesized ZnONPs was predominantly hexagonal in shape even though nanorod-shaped structures were also observed in chemically synthesized ZnONPs. The UV–Vis spectrum of ZnONPs__PPE and ZnONPs_CE showed which are the typical characteristic peaks of ZnO NPs and confirmed the formation of ZnONPs. Further, the biosynthesized ZnONPs_PPE and ZnONPs_CE have proven their compatibility in the MTT assay using VeroE6 cells with higher cell viability compared to ZnONPs_Chem, validating their safety in a concentration-dependent manner. However, they could not be verified as antibacterial agents against the selected strains at the applied concentrations. Overall, the application of the uncalcination method of PPE and CE-derived ZnONPs was found convenient in terms of physiochemical and biocompatibility properties, in comparison with calcined derived ZnONPs, which needs more steps and higher temperatures. This study provides initial insights towards the promising action of green-mediated synthesis of ZnO nanoparticles for safe and efficient applications in agriculture. Yet, a further comprehensive study will be needed for the nanofabrication of ZnONPs using a well-controlled synthesis process at room temperature.

## Data Availability

All data generated or analyzed during this study are included in this published article.

## Sample Availability

Samples of the nanoparticles are available from the authors.
